# Effects of elastic therapeutic taping on reducing drooling in children with neurological disorders: a systematic review of randomized controlled trials

**DOI:** 10.1186/s11689-024-09584-3

**Published:** 2024-12-19

**Authors:** Krystal Tsz Ting Lam, Alex Tsz Wai Hung, Kendy Lau, Eric Kam Pui Lee

**Affiliations:** 1 Jockey Club School of Public Health and Primary Care, Faculty of Medicine, The Chinese University of Hong Kong, Shatin, Hong Kong SAR, China; 2 Li Ping Medical Library, The Chinese University of Hong Kong, Shatin, Hong Kong SAR, China

**Keywords:** Kinesio-taping, Elastic therapeutic taping, Drooling, Neurological disorders

## Abstract

**Background & aims:**

Effective treatment for anterior drooling in children with neurological disorders can lead to improved social interactions, reduced physical complications such as perioral infections, and enhanced quality of life for both patients and their parents. Elastic therapeutic taping (ETT) has emerged a novel intervention for drooling, but its evidence was limited. This study systematically reviewed the effectiveness of ETT on reducing anterior drooling in children with neurological disorders.

**Methods:**

Multiple electronic databases, such as Ovid MEDLINE, Embase, and Cochrane Library were searched from inception till 30th October 2024. Randomized controlled trials (RCTs) were included if they: (a) used ETT as a treatment for drooling or swallowing difficulties; (b) included participants aged < 18 years old; (c) included participants with anterior drooling and neurological disorders; (d) compared effects of ETT alone or combined with other treatments (e.g. oral motor therapy (OMT)) with no taping, sham taping or other treatments, and (e) published in English. The Cochrane Risk-of-Bias tool was used to assess risk of bias for the included studies.

**Results:**

Seven parallel-arm RCTs, which were conducted in South/southwest Asia, Africa, South America and Middle East, were included. In total, 220 children aged 1 to 11 were included, of which 97 received solely ETT in 4 studies, while 24 received ETT plus OMT in 2 studies. ETT combined with OMT was more effective in reducing drooling in the included 2 RCTs, though the results of ETT alone were inconsistent, likely due to heterogeneity observed in control conditions, application methods, and outcome measures. No side effects were reported in all studies.

**Conclusions:**

This review suggests that ETT combined with OMT is effective in reducing drooling in children with neurological disorders, with no evidence of side effects.

**Trial registration:**

(PROSPERO no.: CRD42023488664)

## Introduction

Anterior drooling refers to the uncontrolled loss of saliva from the mouth and is considered developmentally abnormal when it persists in children above the age of 4 [1]. It is prevalent in children with neurological disorders such as cerebral palsy (CP), neuromuscular disorders, intellectual disability, and Rett syndrome, affecting around 10% to 58% of these children [2, 3]. Mechanisms of anterior drooling in these patients include oral motor dysfunctions such as inadequate lip seal, swallowing difficulties, intraoral sensitivity disorders, poor head control, abnormal gag reflex, and tongue thrust [3, 4]. Furthermore, drooling can lead to compromised social interactions, peer avoidance, dissatisfaction with appearance, speech and eating problems and physical complications such as skin irritation and perioral infections [5]. Treating drooling in these children can reduce these complications, improve the quality of life for both patients and their caregivers, and decrease healthcare resource utilization [1].

A variety of interventions are available for anterior drooling, including behavioral strategies, oral motor exercises, pharmacological treatments, botulinum toxin A injections, and surgery [1]. Treatments are typically chosen based on patients' needs, following a progressive therapeutic paradigm in which conservative approaches precede pharmacological interventions and surgeries [1]. However, there are downsides to all existing treatments [6, 7]. For instance, pharmacological interventions inherently carry the risk of side effects such as irritation and constipation; and surgical methods are invasive and are associated with complications such as aspiration pneumonia [7, 8]. However, the effectiveness of conservative treatments such as behavioral interventions and oral motor therapy (OMT) in these children are not well-established [9, 10].

Elastic therapeutic taping (ETT), also known as kinesiotaping, has emerged as a novel and non-invasive modality of treatment for drooling. ETT is an appealing treatment option due to its ease of use (as patients or their caregivers can apply ETT long-term after proper education), accessible (being typically affordable) and has the potential to address underlying oral motor dysfunctions without the need for intensive therapy sessions because applying ETT to the orbicularis oris or suprahyoid areas can stimulate cutaneous receptors, improve proprioception, modulate muscle tone, and accelerate muscle re-education through ETT's elasticity and reactive force [11]. A systematic review conducted in 2019 suggested that ETT was a viable and low-cost drooling treatment without adverse effects in children with neurological disorders [12]. However, that review only included quasi-experimental studies and did not include any randomized controlled trials (RCTs) which compared ETT with a control group. Also, screening, data extraction, and quality assessment were not conducted by at least 2 independent reviewers. That review concluded that there were few studies of high methodological quality, and that the effectiveness of ETT was inconclusive. Since our current review was registered in 2023, two relevant new systematic reviews have been published. However, these reviews only include one and two RCTs, respectively. Additionally, the review specific to ETT did not conduct a risk-of-bias assessment for the included RCTs, which is essential for guiding clinical practice and future research [13, 14]. We are also aware that several relevant RCTs were not included in any of these previous reviews.

In the format of population, intervention, control, and outcomes (PICO), this systematic review investigated, in children with neurological disorders including central and peripheral neuropathy and drooling (P), the effectiveness of ETT (either alone or in combination with other interventions) (I), when compared to no taping, sham taping, or other treatments (C), on reducing the severity and frequency of anterior drooling. Only randomized controlled trials (RCTs) were included, as they provide the highest level of medical evidence by eliminating confounding factors and thereby ensuring internal validity. If ETT is found to be effective in reducing drooling, it could serve as a feasible and inexpensive novel treatment option for affected children worldwide. This review also aims to highlight knowledge gaps in existing literature and guide future RCTs.

## Methods

This review complied with the Preferred Reporting Items for Systematic Reviews and Meta-Analyses (PRISMA) guidelines [15], and the protocol was pre-registered with PROSPERO (CRD42023488664).

### Search strategy

We conducted electronic searches in Ovid MEDLINE, Embase, AMED, CINAHL Ultimate, Cochrane Library, Scopus, and Wed of Science from inception till 30th October 2024 to identify all relevant publications. The search was limited to English. A detailed search strategy can be found in Appendix 1. RCTs were also identified by electronic searches of reference lists of relevant reviews and Google Scholar.

### Eligibility criteria

RCTs were included if they: (a) used ETT as a treatment for drooling or swallowing difficulties; (b) included participants aged < 18 years old; (c) included participants with anterior drooling and neurological disorders, including central and peripheral neurological disorders; (d) compared effects of ETT alone or combined with other treatments (e.g. OMT) with no taping, sham taping or other treatments, and (e) published in English. RCTs that used rigid tape as active intervention were excluded. Observational studies, review studies, case reports, and commentaries were excluded.

### Study selection and data extraction

Studies from the search were imported into the Covidence program (*Covidence systematic review software, Veritas Health Innovation, Melbourne, Australia. Available at* www.covidence.org). The Covidence system automatically detected and excluded duplicate studies. Two out of 3 reviewers (EL/KL/AH) conducted abstract screening, full text screening, and data extraction independently. Discrepancies between reviewers were resolved by discussion.

We developed a standardized data extraction form to extract study’s characteristics, including bibliographic details (e.g. author(s), publication year, data collection country), participant characteristics (e.g. sample size, age, sex, diagnosis, drooling frequency and severity baseline), intervention components (e.g. placement of tapes, stretching extent of tapes, frequency and length of treatment), details of control or comparison conditions (e.g. frequency, length), outcome measures (e.g. 5-min drooling quotient (DQ) test, drooling impact scale (DIS), Thomas-Stonell drooling scale (TSDS), drooling severity and frequency scale (DSFS), average daily use of towels), and major findings.

### Risk of bias assessment

Two independent reviewers used the Cochrane Risk of Bias tool version 1 to assess risk of bias for the included RCTs [16]. It encompasses rating criteria for various aspects, including random sequence generation, allocation concealment, blinding of participants and personnel, blinding of outcome assessment, incomplete outcome data, selective reporting, and other sources of bias. All discrepancies between reviewers were resolved through discussion.

## Results

### Study identification

**Fig. 1:**
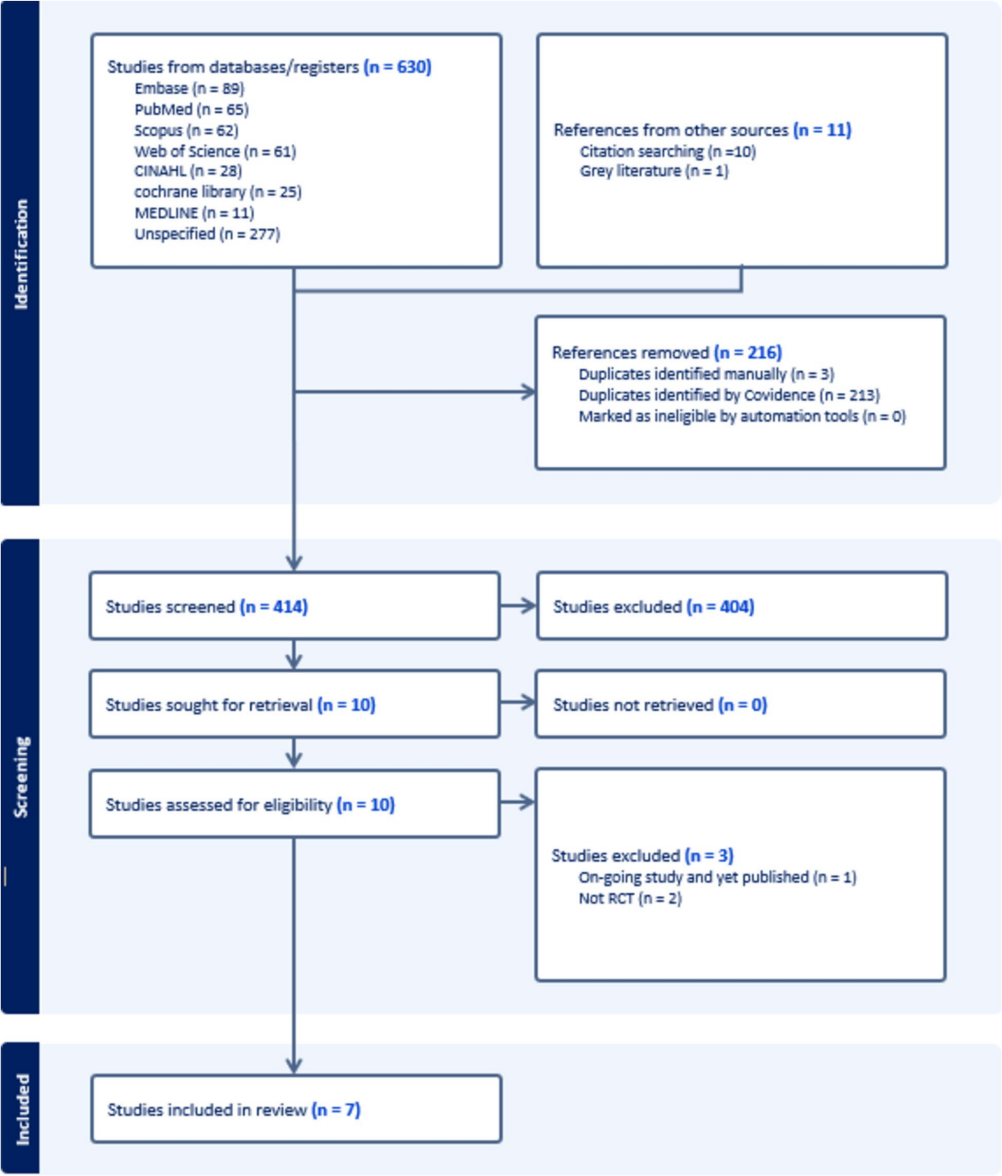
PRISMA diagram

The initial search identified 630 articles. After eliminating duplicates, 414 records remained, of which only 7 articles fulfilled the inclusion criteria and were included (see PRISMA flow diagram Fig. 1). A list of excluded studies after the full text review can be found in Appendix 2.

### Study characteristics

The included 7 studies were all parallel-arm RCTs. The details of the included studies can be found in Tables 1 and 2. All studies were published between 2015 and 2024. The included RCTs were conducted in South/southwest Asia (*n* = 3), Africa (*n* = 1), Middle East (*n* = 2) and South America (*n* = 1). In total, 220 children diagnosed with neurological disorders and drooling were included, in which 89% (*n* = 195) were those diagnosed with CP, and other participants were diagnosed with idiopathic intellectual disability, Down syndrome, childhood stroke, autism, attention deficit hyperactivity disorder, seizure disorder, or developmental delay. The age of the study population ranged from 1 to 11 years old, and 52% were female. Of this study population, 97 received solely ETT in 5 studies, while 24 received a combination of ETT and OMT in 2 studies.

**Table 1:** Characteristics of included studies and main findings

**Source, country**	**Sample**	**Gender**	**Diagnosis**	**Intervention** **Group**	**Control group**	**Control/Comparison**	**Outcome measures**	**Main findings**	**Limitations**^**a**^
Akaltun et al. [17] Turkey	*n* = 101	Female = 41.6% Male = 58.4%	Cerebral palsy (*n* = 101) Drooling (*n* = 75)	*n* = 54 Mean age = 4.2 Drooling (*n* = 41)	*n* = 47 Mean age = 4 Drooling (*n* = 34)	I-shape ETT applied without stretching to the suprahyoid region and not including the origins of mylohyoid and digastric muscles	Rate of presence of drooling	Drooling was found to be significantly improved at six weeks in the ETT group and clinical improvements continued for 18 weeks	Unclear:—sequence generation -blinding of participants and personnel
Awaad et al. [18] Egypt	*n* = 24	Female = 29.2% Male = 70.8%	Spastic cerebral palsy (*n* = 24) Drooling (*n* = 24)	*n* = 12 Mean age = 6.32	*n* = 12 Mean age = 5.9	OMT exercises including perioral sensory stimulation, tapping, tongue pressure, jaw exercises, intraoral stimulation, and training with different sizes of straws	5-min Drooling Quotient (DQ) test Drooling Severity and Frequency Scale (DSFS)	Comparison of pre- and post-treatment mean values of all measured variables showed significant improvement for children of both ETT and OMT groups. The post treatment results showed that OMT is more effective than ETT in decreasing drooling severity and frequency	Small sample size Unclear -sequence generation -allocation concealment -blinding of participants and personnel -blinding of outcome assessor
Mokhlesin et al. [19] Iran	*n* = 18	Female = 44% Male = 56%	Idiopathic intellectual disability (*n* = 8) Cerebral palsy (*n* = 7) Down Syndrome (*n* = 2) Microcephalic (*n* = 1) Drooling (*n* = 18)	*n* = 9 Mean age = 9.8	*n* = 9 Mean age = 10.4	OMT followed by sham taping (without stretching) OMT including brushing and icing of tongue, stroking and tapping of lips, resistance training of lips, tongue resistance training	5-min DQ test Drooling Rating Scale (DRS)	5-min DQ assessment revealed that using ETT plus OMT can produce greater improvement than sham taping plus OMT. However, there was not a statistically difference between the two groups based on parental reports using DRS. Within-group analysis showed that drooling reduced in both groups after the intervention both based on DRS and DQ assessments. Adding taping with and without stretch to OMT can be considered as a complementary method to mitigate drooling in children with intellectual disabilities	Small sample size
Pervez et al. [20] Pakistan	*n* = 20 Mean age = 5.4	Female = 30% Male = 70%	Cerebral palsy (*n* = 18) Childhood stroke (*n* = 2) Drooling (*n* = 20)	*n* = 10	*n* = 10	OMT including tapping, massage and rhythmic pressure	Modified Teachers’ Drooling Scale	Both ETT and OMT were effective in the management of drooling severity	Small sample size Unclear -sequence generation -allocation concealment -blinding of participants and personnel -blinding of outcome assessor
Swati [21] India	*n* = 30	Female = 43.3% Male = 56.7%	Non-spastic cerebral palsy (*n* = 10) Spastic cerebral palsy (*n* = 8) Seizure disorder (*n* = 1) Mental retardation /intellectual disability (*n* = 4) Autism (*n* = 4) Down syndrome (*n* = 1) Developmental delay (*n* = 1) Attention deficit hyperactivity disorder (*n* = 1) Drooling (*n* = 30)	*n* = 15 Mean age = 5.91	*n* = 15 Mean age = 4.92 Lost to follow-up (*n* = 2)	OMT including manipulation, vibration, deep pressure and icing	Thomas-Stonell Drooling Scale (TSDS) Drooling Impact Scale (DIS)	Both groups showed significant improvement on drooling in DIS, TSDS, the lip closure measurement and aspect of social stigma. But OMT along with ETT showed more improvement	Small sample size Unclear -sequence generation -allocation concealment -blinding of participants and personnel High risk: blinding of outcome assessor
de Freitas and Leite [22] Brazil	*n* = 37	Female = 46% Male = 54%	Spastic cerebral palsy (n = 31) Non-spastic cerebral palsy (*n* = 6) Drooling (*n* = 37)	*n* = 18 Mean age = 8	*n* = 19 Mean age = 7	Rigid labial tapping with Micropore medical tape	TSDS Number of wipes used daily	Adhesive tapes fixed under the lower lip promoted a reduction in the number of the wipes used to dry saliva and an improvement in both the severity and frequency of drooling	Small sample size Unclear -sequence generation -allocation concealment -blinding of participants and personnel -blinding of outcome assessor
Yilmaz et al. [23] Turkey	*n* = *48* Mean age = 9.28	Female = 45.8% Male = 54.2%	Cerebral palsy (n = 48) Drooling (n = 48)	*n* = 16 Mean age = 9.28	*n* = *32* Mean age = 9.32	Group 1: no taping (*n* = 16) Group 2: Sham taping: I-tape of approximate 5 cm long applied on one cheek without any tension (*n* = 16)	DSFS 5-min DQ test	There was a significant improvement in patients assigned to the intervention group for the DSFS and 5-min DQ at 45 min (p < 0.05)_and 2 days (p < 0.01) after application. No improvement was observed in the control groups. However, there was no significant difference at the endpoint between the intervention and control groups	Small sample size Unclear -sequence generation -allocation concealment -blinding of outcome assessor High-risk: -blinding of participants and personnel

^a^as suggested by [24] even pilot study should have at least 70 participants in a parallel-arm randomized controlled trials

**Table 2:** Characteristics of ETT interventions in included studies

Source	Intervention	Intensity
Akaltun et al. [17]	Y-shaped ETT (Kinesio Tex, Gold; Kinesio UK, Newcastle upon Tyne, UK) on suprahyoid muscles Tail part of the Y strip adhered with 10 to 15% stretching under the mandibular line to the origins of the mylohyoid muscle	2 sessions/week; 6 weeks (12 sessions) ETT applied for 3 days, then removed for one day of resting
Awaad et al. [18]	I-strip ETT applied on orbicularis oris muscle, one above upper lip and one below lower lip, with maximum stretch	45 min/session; 3 sessions/week; 2 months (24 sessions)
Mokhlesin et al. [19]	OMT followed by ETT (A water resistant EPOS TAPE model of Kinesiotape) I-strip ETT applied on orbicularis oris muscle, one above upper lip and one below lower lip, with 10–15% tension I-strip ETT applied on masseter and suprahyoid muscles with 25% tension	5 min/session; 5 sessions/week; 4 weeks (20 sessions) ETT was on the face for 1 h in the first week, and was weekly increased by a half, such that in the last week, it was on the face for 2 h and a half
Pervez et al. [20]	The procedure included cutting 2 I-strip tapes according to the structure of the patient’s lip muscles. While maintaining 10% tension, the tape was applied to the muscles in the corner of the upper lip with the mouth fully open	45 min/session; 5 sessions/week; 1 month (20 sessions)
Swati [21]	OMT followed by ETT I-strip ETT applied on orbicularis oris muscle, one above upper lip and one below lower lip, with maximum stretch	30 min/session; 3 sessions/week; 30 days (12 sessions) ETT was applied every alternate day
de Freitas and Leite [22]	ETT applied under lower lip	3 h daily; 30 days (30 sessions)
Yilmaz et al. [23]	I-bands are applied to both the upper and lower lips, as well as the suprahyoid muscles, utilizing a tension level of 50–75%, with mechanical correction	2 days

As there was considerable clinical and methodological heterogeneity in ETT interventions, control groups and reported outcomes, a meta‐analysis was not feasible. For instance, two studies compared ETT alone with OMT, two study compared ETT with sham taping (one also includes an inactive control group), one study compared ETT with rigid micropore medical tape, two studies compared ETT plus OMT with OMT alone or OMT with sham taping.

Regarding location of ETT, three studies applied ETT specifically to the orbicularis oris muscle, one study targeted the suprahyoid area, one study placed the ETT under the lower lip, one study applied the ETT to orbicularis oris muscle in addition to suprahyoid area, and one study applied the tape to orbicularis oris muscle, masseter muscle, and suprahyoid area. Studies also applied ETT with different tension, but among all, only Mokhlesin et al. [19] described the objective measurement of tension using meter-gauge before ETT application. In terms of intensity of intervention, the frequency of ETT applications ranged from twice a week to daily. Duration of ETT application ranged from 45 min to 3 days. The total number of sessions ranged from 1 to 30 sessions.

In terms of outcome measures, three studies included the 5-min DQ test and also utilized subjective scales to assess the impact of drooling on daily life, specifically the DSFS and/or the Drooling Rating Scale (DRS). De Freitas and Leite [22] and Swati [21] used TSDS, but the former presented the change in the number of participants at different clinical stages, while the latter calculated mean scores. In addition, de Freitas and Leite [22] measured the number of wipes used daily, and Swati [21] used DIS to measure impact of drooling on quality of life. Pervez et al. [20] used modified teachers’ drooling scale. Akaltun et al. [17] is the only one that measured the number of participants with drooling.

### Effects of ETT as a stand-alone treatment

The effectiveness of ETT as a stand-alone treatment was inconsistent across four studies. Akaltun et al. [17] compared the effectiveness of ETT on suprahyoid muscles (intervention) and sham taping (control), which was applied without stretch, in 75 children diagnosed with CP and drooling. Between group analysis indicated a significant improvement in the ETT group at 6 weeks (post-treatment) (*p* < 0.05) and 18 weeks (follow-up period) (*p* < 0.05). In their control group, there was no significant difference in any parameter at 6 and 18 weeks. A different result was illustrated by de Freitas and Leite [22], which applied ETT under lower lips (intervention), as compared with applying rigid micropore medical tape (control), in 37 children with CP and drooling. Their results showed that ETT and micropore taping both significantly reduced the number of wipes used to dry saliva by 40% and 55% respectively (*p* ≤ 0.05) and reduced drooling severity and frequency (% in clinical stage 4 reduced from 61.1% to 11.1% in intervention group, *p* ≤ 0.05, and from 57.8% to 5.6% in control group, *p* ≤ 0.05). They found no significant difference in effectiveness between the ETT and micropore taping but no relevant statistical details were provided. Similarly, Yilmaz et al. reported intra-group improvement in drooling only in ETT group (*n* = 16) at 45-min and 2 days (*p* < 0.05) but not sham taping (*n* = 16) and inactive control groups (*n* = 16) among participants with CP and drooling. However, there is no significant difference in drooling at the endpoint among these 3 groups.

Another two studies looked at the effectiveness of ETT applied to orbicularis oris muscle when compared to OMT. Pervez et al. (*n* = 20) found that ETT and OMT both significantly improved drooling severity with large (pre-test median[IQR]: 7.5[2], post-test median[IQR]: 6[2.25], *p* = 0.004) and medium effect size (pre-test median[IQR]: 7.5[1.25], post-test median[IQR]: 5.5[2], *p* = 0.025) respectively; however, no significant difference was shown between two groups regarding drooling severity (*p* = 0.06). Awaad et al. [18] (*n* = 24) found that DSFS and 5-min DQ test values were significantly lower after treatment in both ETT group (DQ median[IQR] reduced from 28.75[50–17.5] to 21.25[36.87–10]) and OMT group (DQ median[IQR] reduced from 26.25[55–15.62] to 7.5[19.37–0]) (*p* < 0.01), but OMT was superior than ETT in all the two outcome measures (*p* < 0.05).

### Effects of ETT combined with OMT

Using ETT after OMT demonstrated a superior effect in reducing drooling, when compared to OMT alone or with sham taping in the two included RCTs. Swati [21] compared the effect of ETT applied to orbicularis oris muscle after OMT (intervention) to OMT alone (control) in 30 children. Both groups demonstrated significant improvement in DIS (mean score improvement in intervention group = 30 and that in control group = 20, *p* < 0.001), TSDS (pre-test mean of both groups: > 6, post-test mean of intervention group: 4.2, post-test mean of control group: 3.38, *p* < 0.05), lip closure measurement (mean of intervention group reduced from > 10 to < 10 and that of control group reduced from < 8 to ~ 6, *p* < 0.0001) and aspect of social stigma. However, between-group analysis revealed that ETT plus OMT was more effective than OMT alone in lowering DIS values (*p* < 0.05), reducing social stigma (no *p*-value reported), and improving mouth closure (only at first two weeks, *p* < 0.05).

Similarly, Mokhlesin et al. [19] randomly assigned 18 children with intellectual disability to ETT after OMT (intervention) and sham taping after OMT (control). Within-group analysis showed a significant reduction of drooling by both interventions based on DRS (mean ± (SD) of intervention group reduced from 5.67 (1.93) to 3.33 (1.8) and that of control group reduced from 5.44 (2.5) to 3.89 (2.08), *p* < 0.001) and 5-min DQ test (mean ± (SD) of intervention group reduced from 26.22 (11.64) to 16.33 (8.61) and that of control group reduced from 23.66 (11.96) to 17.66 (12.2), *p* < 0.001). ETT plus OMT was more superior than the control when assessed by 5-min DQ test with a moderate effect size (effect size = 0.62, *p* = 0.008). However, no significant difference between groups was detected by parental reports using DRS (*p* > 0.05).

### Side effects of ETT

Although expected side effects of ETT may include skin irritation and allergies, these could be underreported, as only 2 out of the 7 included studies provided safety data. Akaltun et al. [17] reported that none of the children in either ETT or sham taping group had any treatment-related side effects. De Freitas and Leite [22] also found that ETT did not cause choking or bring changes in salivary parameters involved in maintaining oral health, including salivary flow rate, pH and buffer capacity.

### Risk of bias assessment

The summary of risk of bias assessment using Cochrane Risk of Bias tool version 1 can be seen in Table 3. Of the 7 included RCTs, only Mokhlesin et al. [19] showed low risk of bias in all domains. Most studies did not clearly state their methods of sequence generation (6 out of 7), allocation concealment (5 out of 7), blinding of participants and personnel (6 out of 7). Four out of six studies did not clearly state their methods of blinding outcome assessment.

**Table 3:**
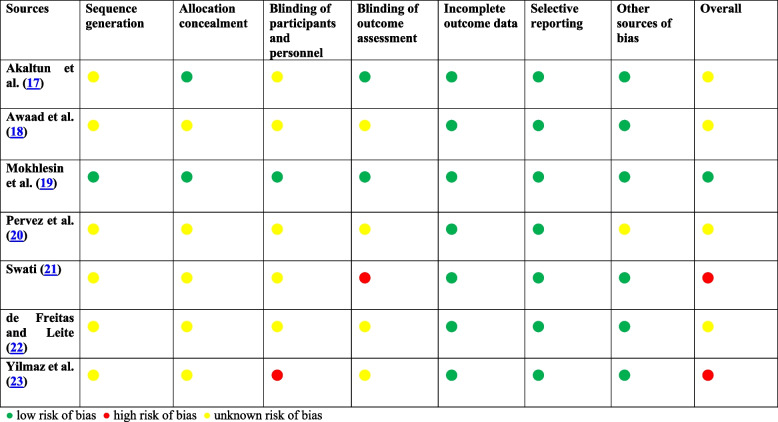
Assessment of risk of bias in RCTs [17–23]

## Discussion

This is the first systematic review of only RCTs that assessed the effectiveness of ETT alone or combined with other treatments compared to no taping, sham taping, or other treatments on reducing drooling in children with neurological disorders. ETT improved drooling in all intra-group comparisons in all 7 included studies. Although the current review included the largest number of RCTs, all existing reviews also consistently concluded that ETT improved drooling in intra-group comparisons with no detected adverse effects [12–14]. Furthermore, ETT combined with OMT was consistently more effective than OMT alone. However, these findings should be interpreted with caution due to the limited number of RCTs to date (*n* = 2). This echoes the findings of a previous study, in which ETT combined with speech therapist-led oral exercises caused rapid improvement in oral motor skills and drooling [25]. When ETT was used as lone treatment, results were inconsistent, possibly due to heterogeneity observed in several aspects of studies.

Although the precise mechanism by which ETT influences muscle activity remains unclear, it has been hypothesized that ETT may enhance proprioceptive stimulation, which could potentially modulate muscle tone and subsequently impact neuromuscular function [26–28]. Individuals with neurological disorders often present with decreased muscle tone, which can subsequently impact the appropriate execution of orofacial movements and swallowing function. Sensory structures play an important role in proprioceptive processing because facial muscles typically lack spindles [29]. In both two studies, ETT was applied on orbicularis oris muscle, which is responsible for controlling lip movements, including closing, protruding, and compressing, to facilitate lip closure [19, 21]. Improved lip closure allows greater containment of saliva within the oral cavity, and the retained saliva may exert pressure on tongue that generates proprioceptive stimuli and triggers swallowing [22, 30]. Besides orbicularis oris muscle, ETT was also applied on masseter muscle and suprahyoid muscles in Mokhlesin et al. [19]. ETT on masseter muscle may facilitate jaw closure, which leads to mouth closure and helps swallowing; on the other hand, ETT on suprahyoid region has been shown to be effective in facilitating hyoid and epiglottic movement during swallowing, and improved swallowing function [17, 31]. When swallowing function improves, saliva swallowing can take place more easily and frequently.

Nonetheless, the effect of ETT alone on reducing drooling has been found to be controversial across studies. This variability in findings may be attributable to the heterogeneity observed in the selection of control conditions or comparisons, location of ETT, tension of ETT, duration of ETT application, and outcome measures employed. The significant differences in ETT application across studies have also been highlighted in the previous systematic review [12–14].

### Selection of control conditions or comparisons

Two of the studies compared ETT on orbicularis oris muscle with OMT targeting a broader range of relevant muscle groups, which may have introduced an inherent bias favoring the OMT, leading to an unfair comparison. In Awaad et al. [18], which found that ETT was less effective than OMT, the OMT included perioral sensory stimulation, tapping, tongue pressure, jaw exercises, intraoral stimulation, and training with different sizes of straws. In Pervez et al. [20], which found that effects of ETT showed no significant differences with that of OMT, the OMT included tapping, finger massage, and rhythmic pressure around the lips, jaw bones, and base of the tongue muscles. Another two studies compared ETT to taping without stretching, but they used different taping materials as control, so their results were not comparable [17, 22]. In Akaltun et al. [17], ETT was compared to ETT applied without stretching; hence, the result would be more attributable to the tension applied. In de Freitas and Leite [22], ETT was compared with micropore medical tape, so the result would be explained by both differences in material use and tension. Even though the use of rigid taping on lips for controlling drooling has not been evaluated in any previous studies yet, it seems that rigid taping is more likely to restrict functional range of motion.

### Location of ETT

Undoubtedly, taping on different muscles can exert different effects on oral motor function and drooling control. All RCTs investigating effects of ETT alone had ETT applied on orbicularis oris muscle, except Akaltun et al. [17], which is the only one study that found superior effect of ETT, and applied ETT on suprahyoid muscles. It is possible that facilitating elevation of hyoid movement to trigger swallowing of saliva is more effective than only improving lip seal, which could lead to pooling of saliva inside the mouth and merely delay the time of anterior leakage. It would be of interest to investigate the effect of ETT alone on orbicularis oris, masseter, and suprahyoid muscles, where Mokhlesin et al. [19] applied ETT in their study, to see if the superior effect of ETT remains without OMT.

### Tension of ETT

There is no uniform standard with respect to tape tensions across all included studies. Typically, ETT can stretch up to 40% of its initial length, so the determination of 0% to 100% of tension was based on this 40% extension of the full length. Although previous studies indicated that different levels of tension did not change muscle strength or function of limbs in healthy individuals, the effect in individuals with neurological disorders is still unknown [32, 33]. De Freitas and Leite [22] suggested that high tension might worsen the drooling in patients with CP co-occurred with Class II malocclusion (anterior open bite). In our review, Akaltun et al. [17] and Mokhlesin et al. [19] were the only two studies that compared ETT applied with 10–25% tension to sham taping without tension using same material. Both of them revealed the superior effect of ETT alone and ETT as an adjunct to OMT. Future research would be required measuring the effect of taping tension in patients with neurological disorders to see how it influences oral motor function and drooling control.

### Duration of ETT

In the three studies that revealed the superior effect of ETT alone or as an adjuvant therapy, the time that ETT stayed on the target location ranged from 2.5 h to 3 days [17, 19, 21]. It is possible that longer application of ETT could bring more influence to muscles, which has been found in previous research on patients with exercise-induced muscle damage [34] and patients with Myofascial Pain Syndrome [35].

### Selection of outcome measures

Similar to the previous reviews on drooling management [6, 12], a great variability is found in the outcome measurements. When outcome measures are highly varied, the results of the studies are not comparable. In the recent systematic review on drooling outcome measures in pediatric disability, 5-min DQ test has been found to be a valid and reliable tool that objectively measures drooling frequency in patients with developmental disabilities [36, 37]. DSFS only had concurrent validity tested, while DRS had no validity or reliability tested [36]. TSDS had criterion validity tested only and was rated ‘doubtful’ in quality appraisal [36]. The number of wipes used daily cannot be reliable as the decision-making on whether or not to change wipes is mostly based on subjective perception [22].

### Research implications

Our results emphasize the need for standardized protocols for ETT applications, regarding site, tension, and duration. Since our findings demonstrated that ETT improved drooling in all intra-group comparisons, future RCTs could directly compare the effectiveness and acceptability of different ETT applications (site, tension, duration). Currently, a head-to-head comparison of these different applications is lacking. For instance, our results suggest that applying ETT to the orbicularis oris muscle, the suprahyoid muscle, or a combination of both can be effective, but their effectiveness has not been directly compared. The future definitive RCT that will determine the effectiveness of ETT should include adequate randomization, blinding of outcome assessors, and standardized application for all patients in the intervention group (e.g., 10–25% tension, as used in 2 of the included RCTs). Additionally, a widely validated outcome measurement, such as the 5-min DQ test, should be adopted, along with an adequate sample size. In fact, the latest review suggests that at least 70 participants are required for a parallel-arm pilot RCT, but only 1 of the included RCTs met this sample size criterion [24].

### Clinical implications

Our results suggest that, despite being reported by only 2 RCTs, ETT as an adjunct to OMT is safe and effective in reducing drooling in children with neurological disorders, with no evidence of harm or side effects. Clinicians should apply ETT judiciously with consideration of individuals’ medical diagnosis, allergic history, oral motor function, swallowing function, resources available, and needs to decide whether ETT should be chosen and in what way it should be applied, including location, tension, duration of application, to achieve the best possible effect. The current acceptability of ETT for managing drooling among patients and their parents is understudied and should be considered when prescribed because applying ETT to the face may carry a stigma associated with being diseased. Additionally, acceptability may vary across different cultures; for example, ETT may be more acceptable in cultures where the face is routinely covered. The included RCTs from Africa, South Asia, and Brazil suggest a high level of acceptability, with dropout rates in ETT group ranging from 0 to 13% (0% in 5 out of 7 RCTs). Furthermore, the application of ETT to the face has also been studied in Europe for patients following facial surgery [38]. Clinicians should also beware of the risk of posterior drooling and saliva aspiration, which can be silent and has not been studied in the existing studies on ETT and drooling yet.

### Strengths and limitations

To date, the present study is the first systematic review of only RCTs that describes effects of ETT on reducing drooling and highlighted no evidence of harm. This review followed PRISMA procedures strictly and was performed without deviations from the initial protocol. A dual reviewer approach was used for screening, data extraction, and risk of bias assessment.

Several limitations in this review can be discussed. Firstly, the included studies are small in sample size, and most are not adequately powered with unclear risk of bias. More well-designed RCTs are required in the future. Secondly, our included studies are from South/southwest Asia, Africa, South America and Middle East. Although no evidence suggests the effects of ETT differ based on geographic or cultural factors, the generalizability of our results to other countries, including the acceptability of ETT, remains unknown. Thirdly, ETT application (intensity, frequency, location) and reported outcomes are very heterogeneous, so meta-analysis could not be conducted. Fourthly, the longest study lasted 2 months only, and only one study measured the prolonged effect of ETT after treatment. Hence, long-term study is lacking and long-term effect of ETT remains unknown. Fifthly, only English studies were included. As recommended by the Cochrane Handbook, searches should not have language restrictions if resources (such as translators) are available. However, this is not feasible for our study, as it is self-funded, and the reviewers can only read English and Chinese. Additionally, evidence suggests that including lower-quality trials published in languages other than English may introduce bias in systematic reviews [39]. Reviews have also concluded that excluding non-English literature does not significantly affect the results, as most medical literature is now published in English [40–42]. Nevertheless, we identified more relevant RCTs than all existing systematic reviews that did not appear to impose language restrictions in their searches [13]. Lastly, the association between effectiveness of ETT and important patients’ demographics could not be investigated due to limited number of studies.

## Data Availability

Data will be available at reasonable request.

## Appendix 1

**Table 4:** Search strategy

1	exp Sialorrhea/ or exp Deglutition/ or exp "Feeding and Eating Disorders"/ or exp "Feeding and Eating Disorders of Childhood"/
2	(Drool* or sialorrhea or swallow* or (oral adj motor) or oromotor or Deglutition* or feeding).ab,ti.
3	1 or 2
4	exp Bandages/ or exp Athletic Tape/
5	(Elastic therapeutic tap* or bandage* or kinesio* tap* or kinesiotap* or k-tap* or athletic tap* or tape or taping or tapes).ab,ti.
6	exp comparative study/ or exp "Randomized Controlled Trials as Topic"/ or exp "Non-Randomized Controlled Trials as Topic"/
7	(randomized controlled trial OR controlled clinical trial OR comparative study).pt.
8	(random OR randomly OR randomized OR randomised OR clinical trial* or controlled trial* or placebo or sham).ab,ti.
9	4 or 5
10	6 or 7 or 8
11	3 and 9 and 10
12	limit to English language

## Appendix 2

**Table 5:** List of excluded studies after full text review

Study	Title	Citation	Main exclusion reason
Sordi et al. 2017	The elastic bandage as a therapeutic resource for the control of sialorrhea: An analysis of its efficacy	Distúrb Comun São Paulo / 2017;29(4):663-671	Not RCT
NCT04252157 2020	Taping for Saliva Control in Cerebral Palsy	N/A	on-going and unpublished study
Kaur, Kaur & Mitra 2022	Effects of sensory motor stimulation and kinesio taping on drooling in spastic cerebral palsy children	Osteoporosis International / 2021;32(SUPPL.1):S277-S278	Not RCT
